# Genome-Wide Characterization and Phylogenetic and Stress Response Expression Analysis of the MADS-Box Gene Family in Litchi (*Litchi chinensis* Sonn.)

**DOI:** 10.3390/ijms25031754

**Published:** 2024-02-01

**Authors:** Jie Yang, Rong Chen, Wei Liu, Xu Xiang, Chao Fan

**Affiliations:** Guangdong Provincial Key Laboratory of Tropical and Subtropical Fruit Tree Research, Key Laboratory of South Subtropical Fruit Biology and Genetic Resource Utilization, Ministry of Agriculture and Rural Affairs, Institute of Fruit Tree Research, Guangdong Academy of Agricultural Sciences, Guangzhou 510640, China; yangjie95222@126.com (J.Y.);

**Keywords:** litchi, MADS-box gene family, phylogenetic analysis, stress response, gene expression

## Abstract

The MADS-box protein is an important transcription factor in plants and plays an important role in regulating the plant abiotic stress response. In this study, a total of 94 MADS-box genes were predicted in the litchi genome, and these genes were widely distributed on all the chromosomes. The LcMADS-box gene family was divided into six subgroups (Mα, Mβ, Mγ, Mδ, MIKC, and UN) based on their phylogenetical relationships with *Arabidopsis*, and the closely linked subgroups exhibited more similarity in terms of motif distribution and intron/exon numbers. Transcriptome analysis indicated that LcMADS-box gene expression varied in different tissues, which can be divided into universal expression and specific expression. Furthermore, we further validated that LcMADS-box genes can exhibit different responses to various stresses using quantitative real-time PCR (qRT-PCR). Moreover, physicochemical properties, subcellular localization, collinearity, and *cis*-acting elements were also analyzed. The findings of this study provide valuable insights into the MADS-box gene family in litchi, specifically in relation to stress response. The identification of hormone-related and stress-responsive *cis*-acting elements in the MADS-box gene promoters suggests their involvement in stress signaling pathways. This study contributes to the understanding of stress tolerance mechanisms in litchi and highlights potential regulatory mechanisms underlying stress responses.

## 1. Introduction

Transcription factors (TFs) are a class of protein molecules that regulate downstream target gene expression with a specific intensity at a specific time and space by binding to *cis*-acting elements, playing irreplaceable roles in biological processes [1]. MADS-box TFs are an ancient gene family found to widely exist in eukaryotes [2]. The name itself originates from the initials of the first four discovered transcription factors in this family, which were MINICHROMOSOME MAINTENANCE 1 (MCM1) in *Saccharomyces cerevisiae,* AGAMOUS (AG) in *Arabidopsis thaliana*, DEFICENS (DEF) in *Antirrhinum majus* and SERUM RESPONSE FACTOR (SRF) in *Homo sapiens* [3]. MADS-box family members typically contain a highly conserved MADS-box domain that consists of approximately 60 amino acids, which is located at the N-terminal region, and this domain can bind CArG-box (CC (A/T) 6GG) sequences in the target gene [4]. Based on the protein domain structure and phylogenetic relationships, the MADS-box gene family is divided into two categories, Type I and Type II [5]. In general, the Type I MADS-box genes contain 1–2 exons and a highly conserved SRF-like MADS domain, also known as M-type genes; they can be further divided into four subgroups: Mα, Mβ, Mγ, and Mδ [6]. The Type II MADS-box genes differ from Type I in that they consist of 6-7 exons, including four domains: MADS (M), the Intervening (I), the Keratin-like (K), and the C-terminal (C), also known as MIKC-type genes; they can be further divided into two subgroups, MIKC^c^ and MIKC*, according to their MIKC structural features [7].

Researchers have elucidated that MADS-box TFs are important regulators in the classic ‘ABCDE’ floral organ development model that reveals their roles in floral organ development [8]. A (*AP1* and *AP2*) and E (SEP) genes are involved in sepal development; A, B (*AP3* and *PI*) and E genes together regulate petal formation; B, C (AGAMOUS) and E genes are responsible for regulating stamen formation; C and E genes are involved in carpel differentiation; C, D (*AGL11*) and E genes are jointly involved in ovule development [9].In addition, the MADS TFs play important roles in many aspects of plant growth and development, including the following: determination of flowering time, *AGAMOUSLIKE GENE 24* (*AGL24*) [10] and Short Vegetative Phase (SVP) [11]; fruit development and ripening, *SHATTERPROOF 1–2* (*SHP1/SHP2*) [12] and FRUITFULL (FUL) [13]; embryo development and lateral root growth, *TRANSPARENT TESTA16* (*TT16*) [14] and *AtAGL21* [15]. Previous research has focused on the function of the MADS-box genes in plant growth and development, while the stress resistance-related function of the MADS-box genes is still not clear.

Adverse environmental conditions, such as drought, low-temperature, and high-temperature, are the primary challenges of plant growth. An unfavorable environment severely limits the geographical distribution and development of plants in nature, and in severe cases, it can cause plant death, thereby leading to a reduction in economic crop production [16]. Adapting to stressed environments, plants have formed a series of complex regulatory networks during long-term evolution, including molecular, cellular, physiological, and biochemical level alterations and stress-related TF regulation [17]. Currently, many studies have been conducted to identify and describe the stress-related genes in various plant species to reveal the complex stress response mechanisms in plants [18,19,20]. In particular, the discovery of abscisic acid (ABA) receptors, progress in understanding the transcriptional and post-transcriptional regulation of stress-responsive gene expression, and studies on hormone interactions under stress have facilitated addressing the molecular basis of how plant cells respond to abiotic stress [21]. Many TFs have been reported to participate in the regulation of stress-related gene expression through ABA-dependent or ABA-independent signaling pathways [22,23]. Recently, it has been reported that the MADS-box TFs are involved in various abiotic stress response processes. For example, in *Arabidopsis*, *AGL91* was regulated by cold stress [24]; in rice (*Oryza sativa*), *OsMADS2*, *OsMADS30*, and *OsMADS55* showed down-regulation in response to dehydration and salt stress, *OsMADS18*, *OsMADS22*, *OsMADS26* and *OsMADS27* showed up-regulation in response to cold stress and dehydration, while *OsMADS87* exhibited high sensitivity to heat treatment [25]; in maize (*Zea mays*), *ZMM7-L* was induced by drought and salt stress, and the germination rates of *ZMM7-L* transgenic plants were lower than that of wild-type plants when exposed to salt treatment, suggesting that *ZMM7-L* might be a negative gene responsive to abiotic stresses [26].

Litchi (*Litchi chinensis* Sonn.), a perennial fruit tree in the Sapindaceae family, has a long history of cultivation in China, dating back over two thousand years [27]. It is highly valued for its economic, nutritional, medicinal, and ecological values, making it a valuable fruit tree crop [28]. The genome-wide identification of MADS-box TFs provides great support for comprehending their biological functions. However, the research on the MADS-box gene family in litchi is limited, with only a few studies focusing on their significant roles in flower organogenesis and floral sex determination [29]. To elucidate the functions of LcMADS-box genes in the stress response, a comprehensive genome-wide study was conducted using a recently published genome database [27]. In this study, we aim to identify the LcMADS-box family members using bioinformatics methods, systematically analyzing their chromosome localization, gene structure, evolutionary relationships, *cis*-acting elements, gene replication, and tissue-specific expression. The expression patterns of LcMADS-box genes under three stress treatments (cold, heat, and drought) were further surveyed. These data help to understand the LcMADS-box genes further, explore the functions of LcMADS-box family members in tissue development and the stress response, and provide a foundation for the exploration of stress-related responses in litchi.

## 2. Results

### 2.1. Identification and Physicochemical Property Analysis of LcMADS-Box Gene Family Members

In the litchi genome, 94 putative LcMADS-box genes were predicted upon combining the results of HMM and conserved domain identification. These LcMADS-box genes were renamed *LcMADS1* to *LcMADS94* based on their distribution across different chromosomes in litchi. Then, we calculated the physical and chemical characteristics of the putative LcMADS-box proteins (Table S1). Specifically, the coding sequence (CDS) sizes of 94 LcMADS-box genes ranged from 228 bp to 1620 bp, and their protein lengths ranged from 75 aa to 540 aa. LcMADS12 had the lowest molecular weight (MW) of 8.44 kDa, while *LcMADS30* had the highest molecular weight of 6066 kDa among these genes. The theoretical isoelectric point (pI) of 94 LcMADS-box proteins ranged from 5.22 to 9.95. A total of 22 LcMADS-box proteins had a theoretical pI of below 7.00, indicating that these proteins were acidic. Except for *LcMADS12*, *LcMADS14*, *LcMADS35*, *LcMADS37*, *LcMADS44*, *LcMADS59*, *LcMADS70*, *LcMADS71*, *LcMADS80*, and *LcMADS93*, the instability index of genes in the rest of the genome was more than 40. The grand average of hydropathicity (GRAVY) values of all 94 LcMADS-box proteins were less than zero, indicating that these proteins were hydrophilic. Except for *LcMADS86*, the number of transmembrane domains in the remaining 93 LcMADS-box proteins was analyzed as 0. Additionally, subcellular localization predictions revealed that *LcMADS12*, *LcMADS14*, *LcMADS51*, *LcMADS63*, *LcMADS71*, *LcMADS77,* and *LcMADS81* were located in the cytoplasm, while the remaining 87 LcMADS-box proteins were located in the nucleus.

### 2.2. Phylogenetic Analysis of LcMADS-Box Gene Family Members

**Figure 1:**
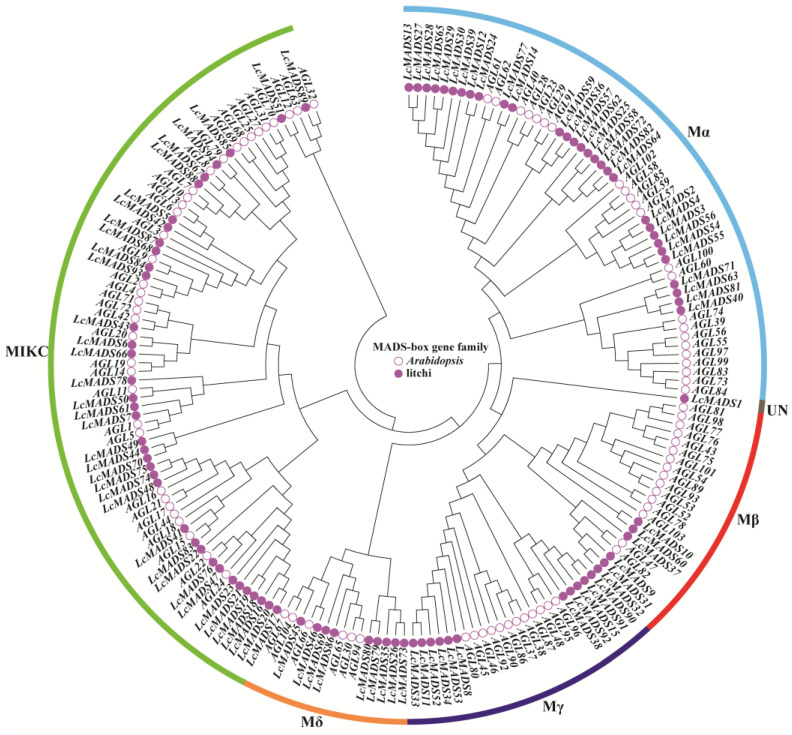
Phylogenetic tree of MADS-box gene family members from litchi and *Arabidopsis*. The filled circles represent the LcMADS-box genes, and the empty circles represent the AtMADS-box genes.

To clarify the evolutionary relationship of MADS-box genes in litchi and Arabidopsis, a phylogenetic tree was constructed based on 94 LcMADS-box proteins and 93 AtMADS-box proteins (Figure 1 and Table S2). According to the classification method of the AtMADS-box family [3], all of these 187 MADS-box genes were grouped into six subgroups (Mα, Mβ, Mγ, Mδ, MIKC, and UN) in the phylogenetic tree. In the LcMADS-box gene family, subgroups Mα, Mβ, Mγ, Mδ, and MIKC contained 30, 6, 11, 9, and 37 members, respectively. It is notable that *LcMADS1* was not classified into any of these subgroups, and therefore, it was grouped into the UN subgroup.

### 2.3. Chromosomal Distribution and Synteny Analysis of LcMADS-Box Gene Family Members

**Figure 2:**
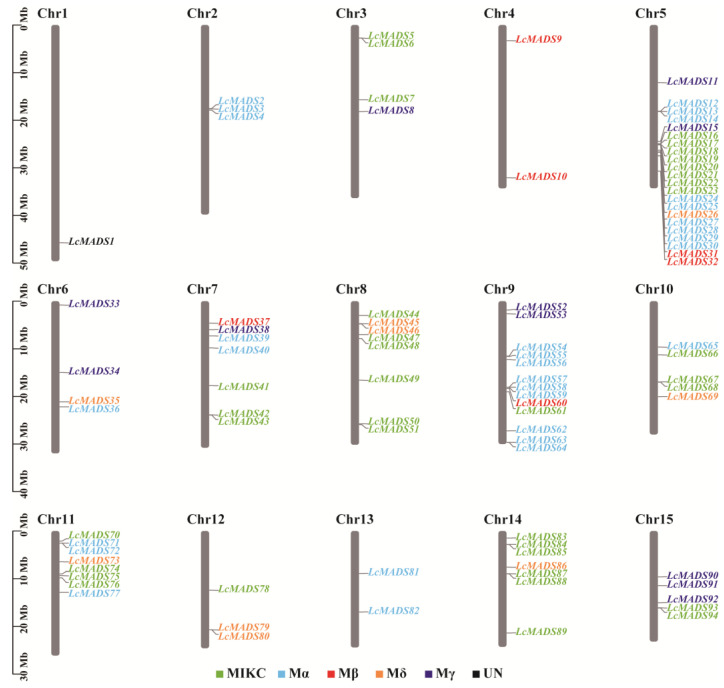
The chromosomal distribution of LcMADS-box genes. The LcMADS-box genes are numbered sequentially from 1 to 15. Different colors represented different groups.

The positional relation of all LcMADS-box genes on the chromosomes is shown in Figure 2. The results showed that 94 LcMADS-box genes were located on 15 different chromosomes, with an uneven distribution of LcMADS-box genes across each chromosome. Chromosome 5 contained the highest number of LcMADS-box genes (22), followed by chromosome 9 with 13 LcMADS-box genes, while chromosome 1 contained the lowest number of LcMADS-box genes (1). Overall, there was no positive correlation between chromosome size and the chromosome that contained the number of LcMADS-box genes. Moreover, most of the LcMADS-box genes contained on a chromosome were grouped into different subfamilies in the phylogenetic relationships within species, revealing that different LcMADS-box genes contained on a chromosome may exercise different functions.

**Figure 3:**
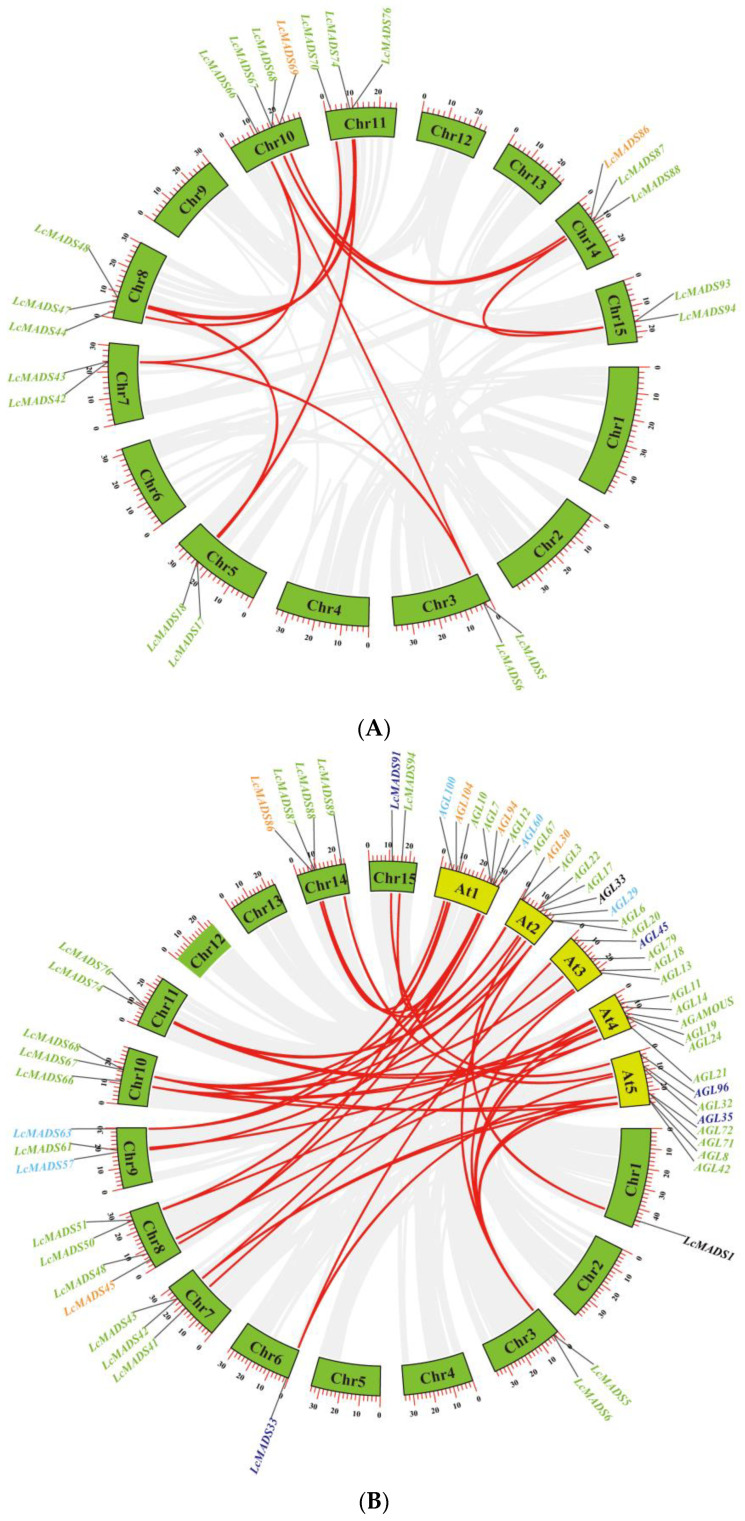
Collinearity analysis of MADS-box gene family members in litchi (**A**) and synteny analysis of MADS-box gene family members between litchi and *Arabidopsis* (**B**). The green and yellow boxes represent the chromosomes of litchi and *Arabidopsis*, respectively. Different gene colors represent the different groups.

To explore the expansion mechanism between the LcMADS-box genes, we examined gene duplication events in litchi. Among the 94 LcMADS-box genes, there were 14 possible pairs of duplicated genes (Figure 3A and Table S3). According to the results of gene duplication analysis, all predicted paralogous genes were segmental duplications (SDs). This indicated that SD was the major driver in the expansion of the LcMADS-box gene family. Ka/Ks (synonymous/non-synonymous) values were calculated using TBtools, and we found that the Ka/Ks ratio varied from 0.0702575 to 0.3105478 (Table S4), indicating that purifying selection plays an important role during gene duplication.

To better explore the evolutionary mechanisms among the MADS-box genes of litchi and *Arabidopsis*, synteny analysis was carried out to identify orthologous gene pairs across the two species. Dual synteny analysis showed that 25 LcMADS-box genes and 33 AtMADS-box genes were orthologous gene pairs, resulting in 44 syntenic relationships between the two species (Figure 3B and Table S5). A total of 18 of the 25 (72%) MADS-box orthologous genes in litchi belonged to MIKC; among the remaining seven, two belonged to Mα, two belonged to Mδ, two belonged to Mγ, and one belonged to UN. Interestingly, there were no orthologs belonging to Mβ.

### 2.4. Gene Structure, Conserved Motif, and Domain Analysis of LcMADS-Box Gene Family Members

**Figure 4:**
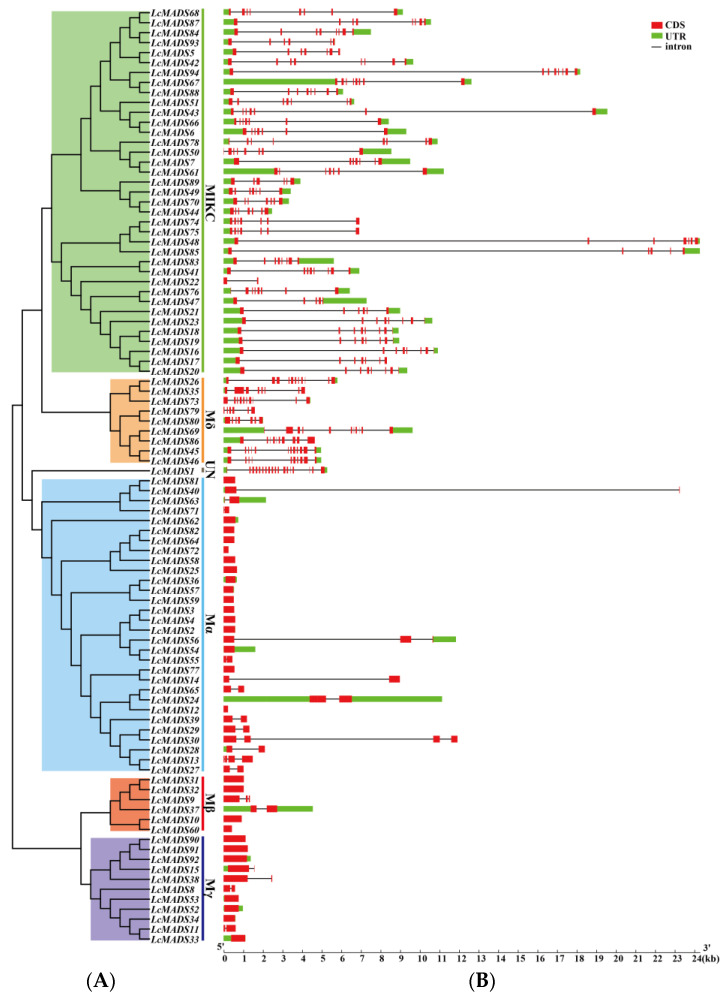

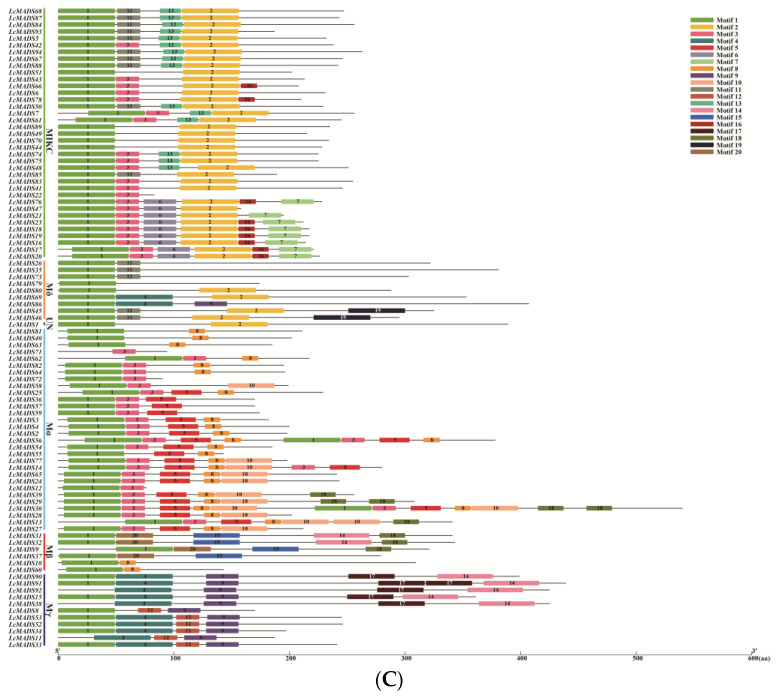
Phylogenetic tree, gene structures and protein motifs in LcMADS-box family members. (**A**) Phylogenetic tree of LcMADS-box genes. (**B**) The structures of the introns and exons and untranslated regions (UTRs) are shown as the black line, red boxes, and green boxes, respectively. (**C**) Twenty conserved motifs in the LcMADS-box proteins are shown, with each small box indicating a motif.

A phylogenetic tree consisting of LcMADS-box gene family members divided into six subgroups was established; this was followed by gene structure and conserved motif analyses (Figure 4). The results of gene structure analysis indicate that the gene structures of 94 LcMADS-box genes were relatively variable, as the exon number ranged from 1 to 18 (Figure 4B). A very striking distribution of introns in LcMADS-box genes was discovered: the subgroup MICK of LcMADS-box genes contained multiple introns, similar to the subgroups Mδ and UN, whereas the remaining three subgroups (Mα, Mβ, and Mγ) had no introns or only one or two introns. A total of 27 intron-free LcMADS-box genes were detected, including 16 genes belonging to subgroup Mα, 4 belonging to subgroup Mβ, and 7 belonging to subgroup Mγ. LcMADS-box genes that were relatively close to each other had similar structures. For example, *LcMADS10*, *LcMADS31*, *LcMADS32,* and *LcMADS60* both contained one exon and had small differences in structure, length, and distribution.

To gain insights into the structural characteristics of LcMADS-box gene family members, we analyzed the conserved motifs according to their phylogenetic relationships. As shown in Figure 4C, a total of 20 conservative motifs were predicted and named from Motif 1 to Motif 20 (Table S6). Among them, Motif 1, Motif 3, and Motif 4 were the MADS domain, and Motif 2 was the K-box domain. The MADS domains were detected in all 94 LcMADS-box genes. Motif 6, Motif 7, Motif 13, and Motif 16 were unique to the members of group MIKC, and they only existed in some of the MIKC members; Motif 9 was unique to the two members of group Mδ; Motif 5 and Motif 10 were unique to Mα; Motif 15 and Motif 20 were unique to Mβ; Motif 12 and Motif 17 were unique to Mγ. MIKC and Mδ shared two similar motifs (Motif 2 and Motif 11); Mδ and Mγ shared two similar motifs (Motif 4 and Motif 9); Mα and Mβ shared two similar motifs (Motif 8 and Motif 18); while Mβ and Mγ only shared one motif (Motif 14). Moreover, all MIKC members had Motif 1, while all Mγ members had Motif 9.

### 2.5. Secondary Structure Prediction of LcMADS-Box Gene Family Members

The secondary structures of LcMADS-box proteins are shown in Table S7. The results showed that 94 LcMADS-box proteins had an alpha helix, extended strand, beta-turn, and random coil. Among these structures, an alpha helix accounted for the largest proportion in 76 of the LcMADS-box proteins, while a random coil accounted for the largest proportion in the remaining 18 LcMADS-box proteins. In addition, alpha helix > random coil > extended strand > beta-turn was predicted in 76 of the LcMADS-box proteins, and random coil > alpha helix > extended strand > beta-turn was predicted in 18 LcMADS-box proteins.

### 2.6. Cis-Acting Elements Prediction of LcMADS-Box Gene Family Members

**Figure 5:**
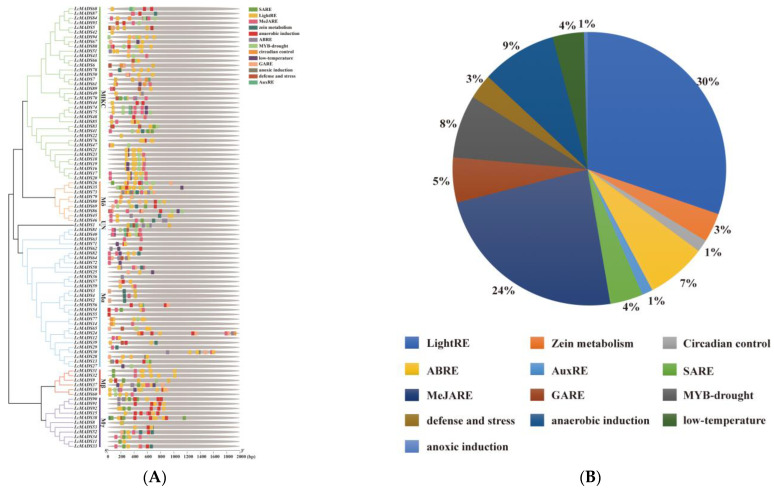
The *cis*-regulatory elements in the promoter region of LcMADS-box gene family members (**A**) and their proportions (**B**). SARE (salicylic acid response element), LightRE (Light response element), MeJARE (methyl jasmonate response element), ABRE (ABA response element), GARE (gibberellin response element), and AuxRE (auxin response element).

The analysis of *cis*-acting elements predicted 13 major *cis*-acting elements in the promoter sequences of the LcMADS-box genes (Figure 5A and Table S8). Among the promoter regions of all 94 LcMADS-box genes, hormone-related *cis*-regulatory elements (such as salicylic acid, methyl jasmonate, abscisic acid, gibberellin, and auxin) accounted for the largest proportion (41%), and were grouped in the first category (Figure 5B). Among the eight *cis*-regulatory elements, plant growth and development elements (such as light responsiveness, zein metabolism, and circadian control) were the second largest category (34%). The remaining stress-related elements (such as drought, low-temperature, anaerobic induction, anoxic induction, and defense/stress) were the third largest category (25%). There were 39 genes involved in drought induction, including 19 members of the MIKC group, 7 members of Mδ, 6 members of Mα, 3 genes in Mβ, 3 genes in Mγ, and 1 gene in UN. It can be seen that the genes in the MIKC group play an important role in the drought response. The promoter regions of 24 genes had low-temperature responsiveness elements, of which 10, 2, 10, and 2 genes were in MIKC, Mδ, Mα, and Mβ, respectively. A total of 40 genes had anaerobic induction response elements. *LcMADS28*, *LcMADS30,* and *LcMADS73* contained anoxic induction response elements and the promoter regions of 18 genes involved in defense/stress. The results of *cis*-acting elements indicated that LcMADS-box genes can respond to a variety of growth factors, hormones, and stresses, and these elements may directly regulate the stress response ability of LcMADS-box genes under stressful environments.

### 2.7. Protein–Protein Interaction Network of LcMADS-Box Gene Family Members

**Figure 6:**
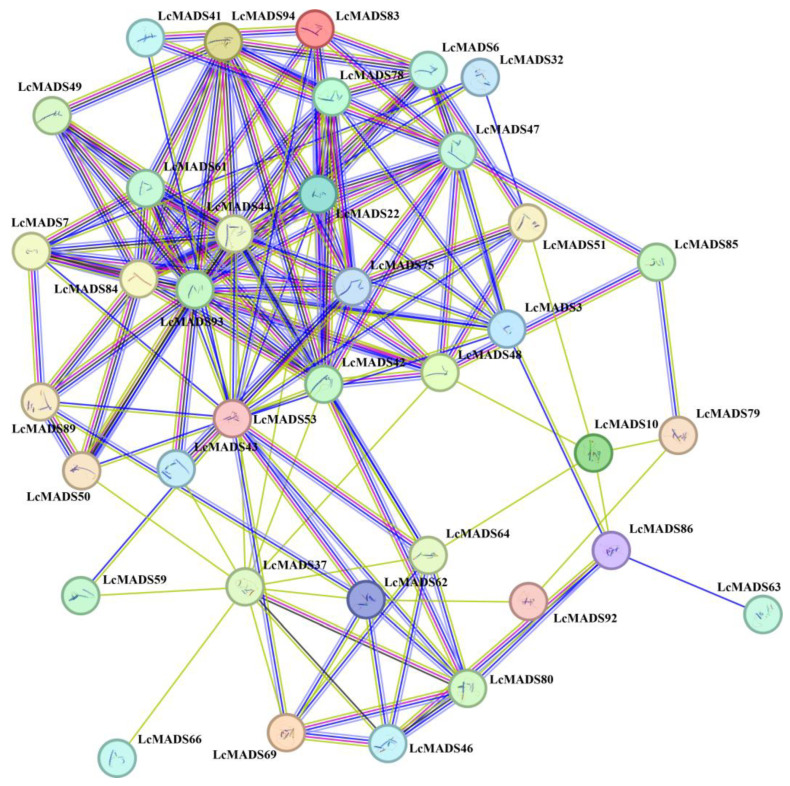
Protein–protein interaction network of LcMADS-box family members. The lines connecting proteins within the PPI network with darker colors and thicker lines indicate higher core PPI values.

To elucidate the potential functions and metabolic pathways of LcMADS-box genes, a protein–protein interaction network was constructed based on the STRING database (Figure 6). The results showed a close interaction among 37 LcMADS-box proteins (*LMADS3*, *LMADS6*, *LMADS7*, *LMADS10*, *LMADS22*, *LMADS32*, *LMADS37*, *LMADS41*, *LMADS42*, *LMADS43*, *LMADS44*, *LMADS46*, *LMADS47*, *LMADS48*, *LMADS49*, *LMADS50*, *LMADS51*, *LMADS53*, *LMADS59*, *LMADS61*, *LMADS62*, *LMADS63*, *LMADS64*, *LMADS66*, *LMADS69*, *LMADS75*, *LMADS78*, *LMADS79*, *LMADS80*, *LMADS83*, *LMADS84*, *LMADS85*, *LMADS86*, *LMADS89*, *LMADS92*, *LMADS93* and *LMADS94*), and there were 144 interaction relationships. *LcMADS44* was located at the core of the interaction network and interacted with *LMADS6*, *LMADS7*, *LMADS22*, *LMADS42*, *LMADS48*, *LMADS53*, *LMADS61*, *LMADS75*, *LMADS78*, *LMADS84*, *LMADS93* and *LMADS94*. It is notable that the remaining 57 LcMADS-box proteins did not have any interaction relationship, and these proteins may independently play a regulatory role.

### 2.8. Expression Pattern of LcMADS-Box Gene Family Members in Different Tissues

**Figure 7:**
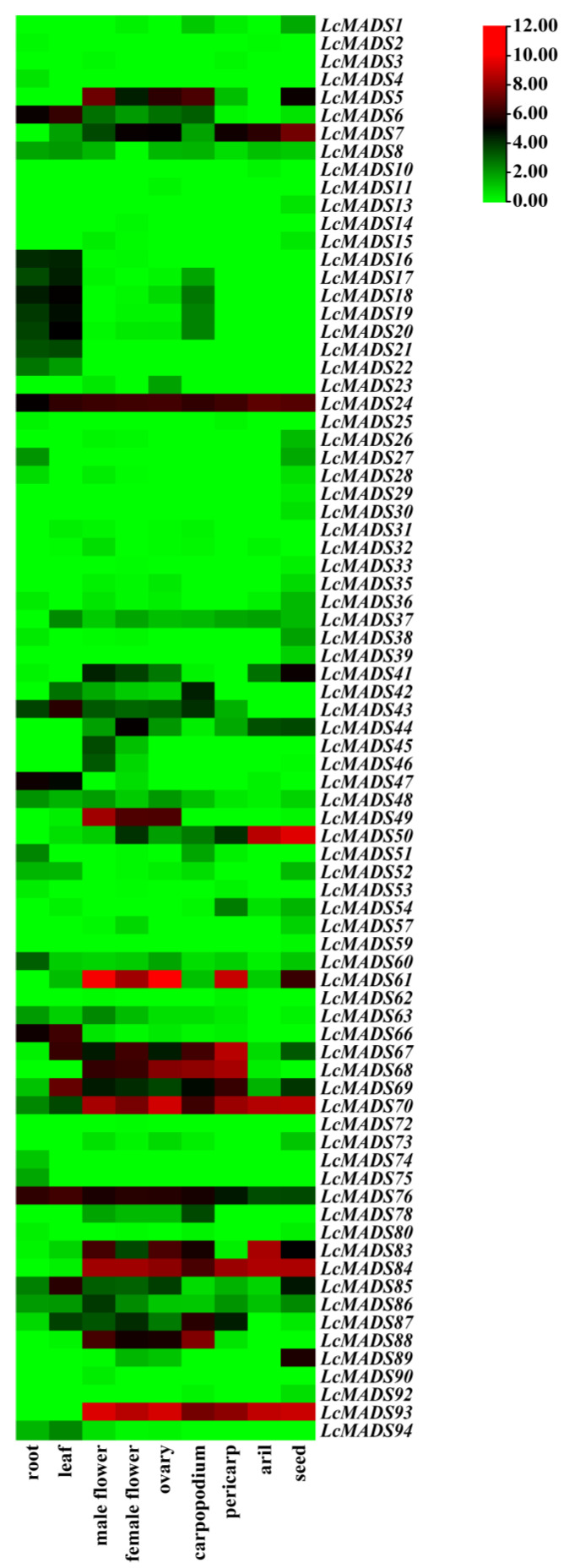
Expression analysis of LcMADS-box gene family members in nine different tissues. The color bar represents the normalized values (log2 FPKM).

Based on public RNA-seq data in the litchi genome database, the expression patterns of LcMADS-box genes in nine different tissues were visualized using heatmap analysis. LcMADS-box genes with FPKM values below 0 in all tissues were excluded. The results showed that 79 of the 94 LcMADS-box genes showed expression in at least one of the nine tissues analyzed in the RNA-seq data, and there were significant differences in tissue-specific expression and expression quantity (Figure 7). For example, 11 LcMADS-box genes, including *LcMADS8*, *LcMADS24*, *LcMADS48*, *LcMADS60*, *LcMADS67*, *LcMADS69*, *LcMADS70*, *LcMADS76*, *LcMADS83*, *LcMADS85,* and *LcMADS86*, were expressed in nine detected tissues; 16 LcMADS-box genes, including *LcMADS6*, *LcMADS8*, *LcMADS16*, *LcMADS17*, *LcMADS18*, *LcMADS19*, *LcMADS20*, *LcMADS21*, *LcMADS31*, *LcMADS37*, *LcMADS43*, *LcMADS66*, *LcMADS69*, *LcMADS76*, *LcMADS85,* and *LcMADS94*, showed higher expression levels in the leaves than in other tissues; 5 LcMADS-box genes, including *LcMADS13*, *LcMADS29*, *LcMADS30*, *LcMADS39,* and *LcMADS59*, were exclusively expressed in seeds; 13 genes, including *LcMADS5*, *LcMADS24*, *LcMADS49*, *LcMADS61*, *LcMADS67*, *LcMADS68*, *LcMADS69*, *LcMADS70*, *LcMADS76*, *LcMADS83*, *LcMADS84*, *LcMADS88,* and *LcMADS93*, exhibited higher expression in male flowers, female flowers, and ovaries.

### 2.9. Expression Analysis of LcMADS-Box Gene Family Members under Abiotic Stresses

**Figure 8:**
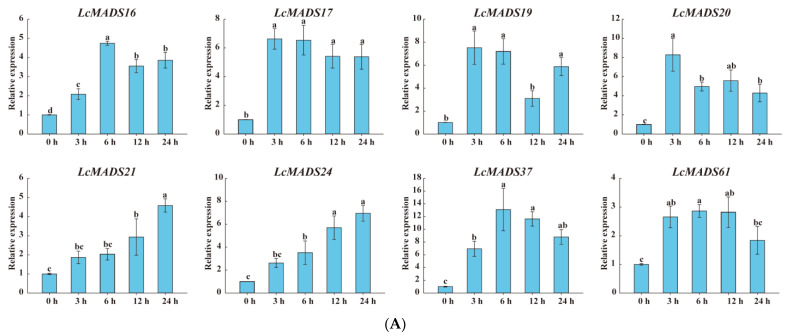

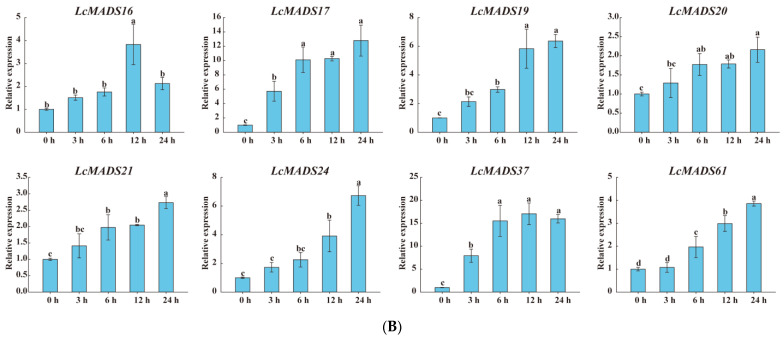

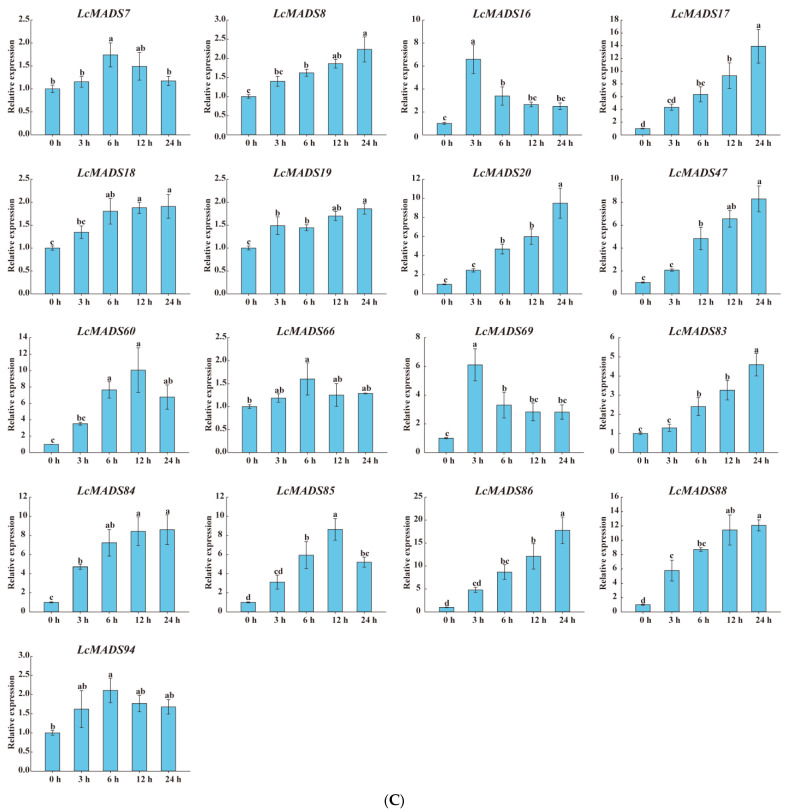
The expression profiles of selected LcMADS-box gene family members in response to different abiotic stresses. Plants were subjected to cold (4.0 ± 1.0 °C), heat (38.0 ± 0.5 °C), and drought stresses (20% (*w*/*v*) PEG6000) for 0, 3, 6, 12, and 24 h. Error bars represent the standard deviation of the mean. (**A**) LT; (**B**) HT; (**C**) PEG. Different letters indicate significant differences, and the same letters represent no significant differences at the 0.05 level.

Considering that the cis-elements responding to various plant hormones, extreme temperature, and drought stress existed in the promoter sequences of LcMADS-box genes, we randomly selected genes expressed in the leaves based on a phylogenetic analysis of the LcMADS-box gene family to examine their expression patterns under cold (8), heat (8) and drought (17) stresses (Figure 8). All of the selected LcMADS-box genes were up-regulated at any time under the stresses, and some differences were extremely significant when compared with the untreated group at time 0 h.

In the case of cold treatment (Figure 8A), after 3 h of treatment, the up-regulation of three LcMADS-box genes (*LcMADS17*, *LcMADS19,* and *LcMADS20*) was the highest among all treatment time points compared with 0 h; it is also notable that three genes (*LcMADS16*, *LcMADS37,* and *LcMADS61*) were obviously up-regulated at 6 h; the remaining two genes (*LcMADS21* and *LcMADS24*) continued to be up-regulated from 1 h to 24 h, indicating that they had not completed their response to cold stress. In the case of heat treatment (Figure 8B), most of the detected LcMADS-box genes were up-regulated to their highest point at 24 h of treatment, while two genes (*LcMADS16* and *LcMADS37*) were highly expressed at 12 h compared with the treatments at the other time points. In the case of drought treatment (Figure 8C), two genes (*LcMADS16* and *LcMADS69*) sharply increased at 3 h, followed by a decrease at 6 h, 12 h and 24 h, compared with the treatment at 3 h; four genes (*LcMADS7*, *LcMADS42*, *LcMADS66* and *LcMADS94*) were significantly up-regulated compared with the treatments at any other time; two genes (*LcMADS60* and *LcMADS85*) were up-regulated to their highest point at 12 h of treatment; the remaining ten genes were found to be significantly up-regulated compared with that of the treatment at any other time point; however, *LcMADS19* transcripts decreased at 6 h. These results suggested that almost all selected LcMADS-box genes are up-regulated in the cold, heat, and drought responses of litchi plants, and these response mechanisms are complex and diverse.

## 3. Discussion

Previous studies have shown that MADS-box transcription factors play a key role in plant growth and development, as well as the response to adverse natural environments [30,31,32]. However, there is little information about the characterization of MADS-box motif-containing proteins in litchi. Therefore, a comprehensive analysis of LcMADS-box gene family members and their expression patterns under various abiotic stresses may be beneficial to further research on the mechanisms of affecting litchi growth and development, as well as their application to litchi molecular breeding.

In this study, a total of 94 MADS-box gene family members were predicted in the litchi genome, named *LcMADS1*, *LcMADS2*, *LcMADS3*, and so on up to *LcMADS94*, depending on their position on the chromosome (Figure 2). This result differs from the 101 MADS-box genes predicted in litchi by Guan et al. [29], which may be caused by the identification methods and screening indicators used for the LcMADS-box gene family. Moreover, the number of MADS-box genes varied considerably among the selected species, i.e., *Arabidopsis* (107 MADS-box genes) [33], rice (75 MADS-box genes) [34], apple (*Malus × domestica*) (146 MADS-box genes) [35], grapevine (*Vitis vinifera*) (90 MADS-box genes) [36], and pear (*Pyrus bretschneideri*) (95 MADS-box genes) [37], which may be related to events such as genome or gene duplication or differences in genome size [2,38,39]. Many MADS-box proteins are subcellularly localized; in fact, most of them are located in the nucleus, such as *AGL15* [40], *AGL24* [41] and *AGL61* [42] in *Arabidopsis*, as well as *OsMADS22*, *OsMADS47*, and *OsMADS50* [43] in rice. Here, most of the LcMADS-box proteins were predicted to be located in the nucleus, as expected. The LcMADS-box genes were divided into Type I (56) and Type II (37). The results of gene structure analysis indicated that the Type I MADS-box gene family members of litchi mostly contained one exon, and their structure was relatively simple. In contrast, the Type II MADS-box gene family members mostly contained 2 to 18 exons, and their structure was more complex than that of Type I. This is similar to the gene structure in pear [37] and wheat (*Triticum aestivum*) [44], further proving that the gene structure is relatively conserved. In general, the K-box domain only exists in the MIKC group [45], but Motif 2 was present in some Mδ members (*LcMADS45*, *LcMADS46*, *LcMADS69,* and *LcMADS80*). It is interesting that Mδ members of Type I genes were also treated as Type II genes in *Arabidopsis* and rice [46]. Xu et al. (2014) believed that the MIKC members lost the K-box while retaining more introns, making them become Mδ genes; with the further loss of introns, Mδ genes became Type I members (Mα, Mβ, and Mγ) with shorter sequences and fewer introns [6].

Previous studies have suggested that MADS-box proteins are classified into five subgroups in *Arabidopsis*, including Mα, Mβ, Mγ, Mδ, and MIKC [47]. With reference to the classification of *Arabidopsis* subgroups, LcMADS-box proteins were categorized into six subgroups (Figure 1), with one gene (*LcMADS1*) in UN, as seen in cacao (*Theobroma cacao*) [3]. The gene structures were similar among the MADS-box genes of the same subgroup, such as *RhMADS7*, *RhMADS9*, *RhMADS10*, *RhMADS31,* and *RhMADS34* [47] in rhododendron (*Rhododendron hainanense*). In this study, most of the LcMADS-box genes had similar structures in the same subgroup, as anticipated. However, a few genes (such as *LcMADS9* and *LcMADS37*) within the same group were structurally different from the other genes, which indicated that these genes had gained multiple introns during LcMADS-box gene family diversification. Furthermore, gene duplications play a key role in the evolution of genomes and genetic systems [48]. The duplicate genes in the litchi genome were analyzed, and 14 pairs of duplicate genes were found (Table S3). A total of 13 pairs of genes had Ka/Ks ratios < 1 among these 14 pairs of LcMADS-box genes, suggesting that most of the LcMADS-box genes had undergone extensive purifying selection.

The *cis*-acting elements of the gene promoter regions are involved in the regulation of gene expression; more precisely, they can regulate the precise initiation and transcriptional efficiency of gene transcription by forming specific binding with transcription factors [49]. In this study, the promoter sequences of the LcMADS-box genes predicted multiple *cis*-acting elements related to plant growth, hormone response, and abiotic stress (Figure 6). This result is consistent with research reports on moso bamboo (*Phyllostachys edulis*) [50] and barley (*Hordeum vulgare*) [51], indicating that the promoter regulatory elements of the MADS-box genes have a certain conservatism among different species. Many studies have demonstrated that abscisic acid, gibberellin, and methyl jasmonate play important roles in plant adaptation to abiotic stress [52,53,54]. Hormone response elements (ABRE, GARE, and MeJARE) are stimulated by corresponding hormone signals and regulate the expression of genes related to stress responses, thereby improving plant adaptability to adverse environments [55]. In this study, 82 of the 94 LcMADS-box genes, excluding *LcMADS8*, *LcMADS11*, *LcMADS27*, *LcMADS39*, *LcMADS42*, *LcMADS44*, *LcMADS53*, *LcMADS55*, *LcMADS66*, *LcMADS68*, *LcMADS69*, *LcMADS71,* and *LcMADS77*, contained varying numbers of hormone-related *cis*-regulatory elements (abscisic acid, gibberellin and methyl jasmonate). In total, 24 LcMADS-box genes, including *LcMADS10*, *LcMADS16*, *LcMADS17*, *LcMADS19*, *LcMADS20*, *LcMADS21*, *LcMADS23*, *LcMADS24*, *LcMADS25*, *LcMADS27*, *LcMADS35*, *LcMADS37*, *LcMADS40*, *LcMADS57*, *LcMADS61*, *LcMADS62*, *LcMADS67*, *LcMADS71*, *LcMADS72*, *LcMADS74*, *LcMADS75*, *LcMADS81*, *LcMADS82,* and *LcMADS86*, contained low-temperature elements. Lastly, 39 LcMADS-box genes, including *LcMADS1*, *LcMADS8*, *LcMADS9*, *LcMADS10*, *LcMADS13*, *LcMADS16*, *LcMADS17*, *LcMADS18*, *LcMADS19*, *LcMADS20*, *LcMADS23*, *LcMADS26*, *LcMADS27*, *LcMADS34*, *LcMADS35*, *LcMADS40*, *LcMADS41*, *LcMADS43*, *LcMADS47*, *LcMADS53*, *LcMADS60*, *LcMADS65*, *LcMADS66*, *LcMADS68*, *LcMADS69*, *LcMADS73*, *LcMADS74*, *LcMADS75*, *LcMADS77*, *LcMADS79*, *LcMADS80*, *LcMADS81*, *LcMADS83*, *LcMADS84*, *LcMADS85*, *LcMADS86*, *LcMADS88, LcMADS93,* and *LcMADS94*, contained drought-related elements. These results show that LcMADS-box gene family members may play essential roles in the growth and development as well as the stress response of litchi.

Studies have shown that the MADS-box genes play fundamental roles in developmental control and signal transduction processes by forming homologous or heterologous complexes [7]. An analysis of the protein–protein interaction network of the LcMADS-box gene family showed that there was interaction among the LcMADS-box members, indicating that they might jointly regulate the development and abiotic stress response of litchi by forming heterologous complexes. We also found that *LcMADS44* occupied a vital position in the protein interaction network, indicating that *LcMADS44* plays a crucial role in the regulation of the development and abiotic stress response of litchi. Tissue-specific expression analysis indicated that *LcMADS44* was not found to be expressed in the leaves, and it was not analyzed in subsequent abiotic stress expression tests.

So far, as we know, the expression of MADS-box genes exhibits a great difference among different species as well as within the same species; for example, in the root, leaf, and female spikelet of maize [56], and the root, leaf, and flower of dandelion (*Taraxacum mongolicum*) [32]. In plum blossom (*Prunus mume*), most MADS-box genes were predominantly expressed in the flowers and fruits, while they were barely or not expressed in the stems and leaves [6]; in wheat, 18 of the 28 detected MADS-box genes were not expressed in the roots, stems, leaves, grains, and spikes, while the remaining 10 genes were expressed in only one to four investigated tissues [44]; in lotus (*Nelumbo nucifera*), 7 genes (*NnMADS1*, *NnMADS2*, *NnMADS4–8*, *NnMADS10*, *NnMADS11*, *NnMADS16,* and *NnMADS17*) were preferentially expressed in floral organs, while *NnMADS41* was specifically expressed in the rhizome internode, and NnMADS43 was leaf- and petiole-specific [39]. Upon analyzing these RNA-seq data, we found that some LcMADS-box genes exhibit tissue-specific expression (Figure 7). Among them, *LcMADS13*, *LcMADS29*, *LcMADS30*, *LcMADS39,* and *LcMADS59* were exclusively expressed in litchi seeds, which may be related to seed development or growth regulation; *LcMADS5*, *LcMADS24*, *LcMADS49*, *LcMADS61*, *LcMADS67*, *LcMADS68*, *LcMADS69*, *LcMADS70*, *LcMADS76*, *LcMADS83*, *LcMADS84*, *LcMADS88,* and *LcMADS93* were preferentially expressed in male flowers, female flowers and ovaries, which may be related to flower differentiation and development. In addition, 15 genes (*LcMADS9*, *LcMADS12*, *LcMADS34*, *LcMADS40*, *LcMADS55*, *LcMADS56*, *LcMADS58*, *LcMADS64*, *LcMADS65*, *LcMADS71*, *LcMADS77*, *LcMADS79*, *LcMADS81*, *LcMADS82,* and *LcMADS91*) were not found to be expressed in any of the tissues tested, and their expression may not be involved in the tested organs of litchi. In conclusion, these results indicate that the LcMADS-box gene family is widely involved in the growth and development of litchi.

Most of the existing studies have been focused on exploring the role of MADS-box genes in flower organ development, with less focus on exploring their responses to various abiotic stresses [57,58]. However, some studies have found that MADS-box genes are actively involved in the abiotic stress response processes [57]. We found that most of the LcMADS-box genes contain multiple hormone-related and stress-related responsive elements, such as *LcMADS16*, which had methyl jasmonate elements, low-temperature elements, and drought elements, and *LcMADS37,* which had abscisic acid elements, methyl jasmonate elements, gibberellin elements, and low-temperature elements, thus warranting further study. Based on the results of the phylogenetic relationships, *cis*-acting elements identification, and heatmap analysis, we selected 8, 8, and 17 LcMADS-box genes that responded to cold, heat, and drought stresses, respectively (Figure 8). Interestingly, we found that all of the detected LcMADS-box genes were up-regulated at all the treated time points, compared with the untreated control; this was consistent with the prediction that the promoter regions contained a large number of hormones and stress response elements. Additionally, the results are consistent with BrMADS genes that respond to cold and drought treatment [59]. Together, these results implied that some LcMADS-box genes actively participated in the responses to various abiotic stresses, which might aid in the selection of suitable candidate genes from the LcMADS-box family for further functional characterization.

## 4. Materials and Methods

### 4.1. Plant Materials and Treatment

‘Feizixiao’ is an early-maturity variety of litchi that has the widest planting areas and the most mature cultivation technology in China [60]. In this study, the annual litchi variety ‘Feizixiao’ was selected as the experimental material. The plant materials were grown in pots (size 26.5 cm × 17.5 cm × 21 cm) with sandy red soil, peat soil, and coconut bran silk (a volume ratio of 3:1:1), with pH values between 5.5 and 6.5 and were maintained in a greenhouse. The experiments were carried out in April 2023 at the Institute of Fruit Tree Research, Guangdong Academy of Agricultural Sciences (113°22′41.200″ E, 23°9′32.418″ N). The litchi seedlings with 25 leaves were selected, and 45 seedlings at the same developmental stage were subjected to three abiotic stress treatments. For cold stress, 15 seedlings were transferred to an incubator at 4.0 ± 1.0 °C under 16 h day/8 h night; for heat stress, 15 seedlings were transferred to an incubator at 38.0 ± 0.5 °C under 16 h day/8 h night; while for drought stress, 20% (*w*/*v*) PEG6000 was used to water 15 seedlings in a greenhouse. The leaves of the cold-stressed, heat-stressed, and drought-stressed plants were collected at 0, 3, 6, 12, and 24 h intervals. Each time point was applied for 3 seedlings under 3 different treatments, and the upper leaf of each seedling was harvested. A mixture of 3 upper leaves was recorded as 1 biological replicate and repeated 3 times. The collected leaves were immediately frozen in liquid nitrogen and stored at −80 °C for RNA extraction.

### 4.2. Identification of LcMADS-Box Gene Family Members

The Hidden Markov Model (HMM) file of the MADS-box conserved domain (PF00319) was downloaded from the protein family (Pfam) database (https://www.ebi.ac.uk/, accessed on 22 February 2023). Then, the HMM profile was compared against the litchi genome database [27] using the HMMER 3.0 software with an expected value (E-value) of 1 × 10^−5^ to search for the LcMADS-box genes. Moreover, all obtained LcMADS-box protein sequences were further analyzed in the CDD database (https://www.ncbi.nlm.nih.gov/cdd/?term=, accessed on 22 February 2023), InterPro database (https://www.ebi.ac.uk/interpro/search/sequence/, accessed on 22 February 2023) and SMART database (http://smart.embl-heidelberg.de/, accessed on 22 February 2023) to verify the presence of the MADS-box domain. Sequences without the complete domain were removed. Finally, the predicted LcMADS-box gene family members were named based on their positional order on the chromosomes.

### 4.3. Basic Physicochemical Properties of the LcMADS-Box Gene Family Members

ExPASy ProtParam (https://web.expasy.org/protparam/, accessed on 27 February 2023), an online tool, was used to analyze the physical and chemical characteristics of the LcMADS-box protein sequences, including the number of amino acids, molecular weight, theoretical isoelectric point (pI), instability index, aliphatic index and grand average of hydropathicity (GRAVY). The number of transmembrane domains in each LcMADS-box protein sequence was retrieved using the TMHMM 2.0 online tool (https://services.healthtech.dtu.dk/services/TMHMM-2.0/, accessed on 5 March 2023). Moreover, the subcellular location of each LcMADS-box protein was predicted using the PSORT prediction tool (https://www.genscript.com/psort.html, accessed on 10 March 2023).

### 4.4. Phylogenetic Analysis of LcMADS-Box Gene Family Members

The predicted MADS-box protein sequences in *Arabidopsis* were downloaded from The Arabidopsis Information Resource (TAIR) (https://www.arabidopsis.org/, accessed on 28 March 2023). Multiple sequence alignment was performed using the MUSCLE program with default parameters and refined manually in the MEGA 7.0 software. Then, a neighbor-joining (NJ) phylogenetic tree was constructed using the MEGA 7.0 software with the Poisson model, pairwise deletion of gaps, and 1000 bootstrap replicates.

### 4.5. Chromosomal Distribution and Synteny Analysis of the LcMADS-Box Gene Family Members

Chromosome position data of the LcMADS-box genes, including chromosome length, gene start positions, and gene end positions, were obtained from the annotation file of the litchi genome. A chromosome distribution map of the LcMADS-box genes was drawn using the Gene Locatin Visualize from the GTF/GFF plugin in TBtools [61]. The One Step MCScanX plugin with default parameters in TBtools was used to analyze LcMADS-box gene replication events. The collinear relationships within the LcMADS-box genes and with the *Arabidopsis* gene homologous to the LcMADS-box genes were plotted using the Advanced Circos plugin in TBtools. In addition, the ratio between the non-synonymous substitution rate (Ka) and the synonymous substitution rate (Ks) of two protein-encoding genes was calculated using the Simple Ka/Ks Calculator (NG) plugin in TBtools, which can be used as an indicator of nucleic acid molecular evolution to determine whether there is a selection pressure on the protein-encoding genes.

### 4.6. Gene Structure and Conserved Motif Analysis of the LcMADS-Box Gene Family Members

The genome and coding sequences of each LcMADS-box gene were obtained from the litchi genome database. The GSDS 2.0 server (http://gsds.gao-lab.org/, accessed on 8 April 2023) was used to construct the gene structure map. The conserved motifs in the LcMADS-box proteins were determined using the MEME online tool (https://meme-suite.org/meme/tools/meme, accessed on 8 April 2023). The predicted number of maximum motifs was 20, and the remaining parameters were the default values. Then, a conserved motif of the LcMADS-box proteins was visualized using the Visualize Motif Pattern (from meme.xml/mast.xml) plugin in TBtools. Moreover, the Conserved Structural Domains Database (CDD) database in NCBI was used for structural domain analysis to determine whether motifs belonged to the MADS-box domain.

### 4.7. Secondary Structure and Cis-Acting Element Prediction of the LcMADS-Box Gene Family Members

The SOPMA online tool (https://npsa-prabi.ibcp.fr/cgi-bin/npsa_automat.pl?page=npsa-_sopma.html, accessed on 15 April 2023) was used to predict the secondary structure of the LcMADS-box proteins. The 2000 bp sequence upstream of the start codon of each LcMADS-box gene was extracted from the litchi genome database and then submitted to the PlantCARE online server (http://bioinformatics.psb.ugent.be/webtools/plantcare/htm-l/, accessed on 20 April 2023) for *cis*-acting element prediction. Finally, the predicted *cis*-acting elements were classified according to their regulatory functions, and the predicted results were visualized using the HeatMap plugin in TBtools.

### 4.8. Protein–Protein Interaction Network of LcMADS-Box Gene Family Members

Protein–Protein interaction (PPI) networks are made up of different proteins that may interact with each other to participate in various aspects of life processes, including biological signal transduction, gene expression regulation, energy and material metabolism, and cell cycle regulation. The STRING database (https://cn.string-db.org/, accessed on 5 May 2023) was used to generate an interaction network model of the LcMADS-box proteins. The species parameter was set to *Arabidopsis*, and the species confidence was 0.40.

### 4.9. Expression Profile of the LcMADS-Box Gene Family Members Based on the Transcriptome Database

RNA-seq data of each LcMADS-box gene were downloaded from the litchi genome database, including seed, aril, root, leaf, male flower, female flower, ovary, carpopodium, and pericarp. These expression data were gene-wise normalized, and the heatmap was drawn using the HeatMap plugin in TBtools. The red color in the heatmap represents the upregulation of the LcMADS-box genes, while green represents downregulation.

### 4.10. qRT-PCR Analysis of the LcMADS-Box Gene Family Members

Total RNA was extracted from all of the leaf samples using the Plant Total RNA kit with Dnase I enzyme (SIMGEN, Hangzhou, China), and then the NanoDrop-2000c-type micro-spectrophotometer (Thermo Fisher Scientific, Guangzhou, China) was used to detect the concentration and purity of the total RNA. cDNA was synthesized using the cDNA First Strand Synthesis kit (SIMGEN, Hangzhou, China) following the manufacturer’s instructions. The coding sequences of 21 selected LcMADS-box genes were obtained from the litchi genome database. The Batch q-PCR Primer Design plugin in TBtools was used to design the specific primers, and Oligo 7.0 software was used to verify the specificity of the primers. The *Actin* gene was selected as the internal reference gene [62], and then the corresponding primers were sent to Guangzhou Tsingke Biotech Co., Ltd. (Guangzhou, China) for synthesis. Detailed primer sequence information can be found in Table S9. Next, real-time quantitative PCR (qRT-PCR) was performed using the 2 × SYBR Green PCR Mix kit (SIMGEN, Hangzhou, China) in a QuantStudio^TM^ 3 Real-Time PCR system (Thermo Fisher Scientific, Guangzhou, China). Details of the procedures are as follows: Stage 1, 95 °C for 2 min for 1 cycle; Stage 2, 95 °C for 20 s, 60 °C for 20 s, and 72 °C for 30 s for 40 cycles (scan); Stage 3, the melting curve ran from 65 °C to 95 °C for 10 s with a ΔT = 0.5 °C (scan). Finally, the average values of three repeated qPCR data were calculated using the 2^−ΔΔCT^ method. DPS 9.01 software was used to analyze the significance (*p* < 0.05), and the gene expression patterns were visualized using SigmaPlot 14.0.

## 5. Conclusions

In summary, this study represents the first genome-wide characterization of the MADS-box gene family in litchi. A total of 94 LcMADS-box genes were predicted in litchi and classified into six subgroups. Chromosome mapping revealed that 94 LcMADS-box genes were distributed on 15 litchi chromosomes. SD was found to play a major role in the expansion of LcMADS-box genes. The subgroup MICK of the LcMADS-box gene family had multiple introns, similar to the subgroups Mδ and UN; however, the remaining three subgroups (Mα, Mβ, and Mγ) had no introns or only one or two introns. Genes belonging to the same family exhibited similar gene structures and conserved motif composition. The *cis*-acting element analysis suggested that LcMADS-box genes may be widely involved in the growth, hormonal, and stress responses of litchi. The RNA-seq data results showed that 79 of the 94 LcMADS-box genes exhibited tissue-specific expression. Furthermore, we demonstrated that LcMADS-box genes play a crucial role in stress tolerance. What is more, this study lays the foundation for further research on the function of LcMADS-box gene family members, providing a possible application in molecular breeding in litchi. Moreover, due to limitations in experimental conditions and manpower, this study only randomly selected genes expressed in the leaves for abiotic stress expression analysis. Thus, further research on LcMADS-box genes in other tissues of litchi under abiotic stresses is required.

## Data Availability

The data are contained within the present article and the Supplementary Materials.

## Supplementary Materials

The supporting information can be downloaded at: https://www.mdpi.com/article/10.3390/ijms25031754/s1.
